# Evidence for a Decrease in Transmission of Ebola Virus — Lofa County, Liberia, June 8–November 1, 2014

**Published:** 2014-11-21

**Authors:** Aditya Sharma, Nico Heijenberg, Clement Peter, Josephus Bolongei, Bruce Reeder, Tamba Alpha, Esther Sterk, Hugues Robert, Andreas Kurth, Angela Cannas, Anne Bocquin, Thomas Strecker, Christopher Logue, Antonino Di Caro, Thomas Pottage, Constanze Yue, Kilian Stoecker, Roman Wölfel, Martin Gabriel, Stephan Günther, Inger Damon

**Affiliations:** 1 Epidemic Intelligence Service, CDC; 2 Médecins Sans Frontières, Operational Center Geneva, Voinjama, Liberia; 3 World Health Organization, Monrovia Country Office, Liberia; 4 Lofa County Health Office, Ministry of Health and Social Welfare, Voinjama, Liberia; 5 Médecins Sans Frontières, Operational Center Geneva, Geneva, Switzerland; 6 University of Saskatchewan College of Medicine, Saskatoon, Canada; 7 European Mobile Laboratory Consortium; 8 Robert Koch Institute, Berlin, Germany; 9 Istituto Nazionale per le Malattie Infettive (Lazzaro Spallanzani), Rome, Italy; 10 Laboratoire P4 INSERM Jean Merieux, Lyon, France; 11 Philipps-University Marburg, Institute of Virology, Marburg, Germany; 12 Public Health England, Porton Down, UK; 13 Bundeswehr Institute of Microbiology, Munich, Germany; 14 Bernhard-Nocht-Institute for Tropical Medicine, Hamburg, Germany; 15 Division of High-Consequence Pathogens and Pathology, National Center for Emerging and Zoonotic Infectious Disease, CDC

Lofa County has one of the highest cumulative incidences of Ebola virus disease (Ebola) in Liberia. Recent situation reports from the Liberian Ministry of Health and Social Welfare (MoHSW) have indicated a decrease in new cases of Ebola in Lofa County (1). In October 2014, the Liberian MoHSW requested the assistance of CDC to further characterize recent trends in Ebola in Lofa County. Data collected during June 8–November 1, 2014 from three sources were analyzed: 1) aggregate data for newly reported cases, 2) case-based data for persons admitted to the dedicated Ebola treatment unit (ETU) for the county, and 3) test results for community decedents evaluated for Ebola. Trends from all three sources suggest that transmission of Ebola virus decreased as early as August 17, 2014, following rapid scale-up of response activities in Lofa County after a resurgence of Ebola in early June 2014. The comprehensive response strategy developed with participation from the local population in Lofa County might serve as a model to implement in other affected areas to accelerate control of Ebola.

Liberia is in the midst of the largest outbreak of Ebola to date, with approximately 6,500 reported cases as of October 31, 2014 (2). MoHSW reported 623 cases in an estimated population of 300,000 in Lofa County by the end of October, the third highest cumulative incidence in Liberia (3).The first cases of Ebola in Liberia were reported in March 2014 in Foya (4), a town of approximately 20,000 persons in Lofa County in northern Liberia. After the emergence of Ebola in the county, local government health offices, nongovernmental organizations, and technical agencies developed a comprehensive response strategy in collaboration with communities. The strategy consisted of the following activities: 1) encouraging changes in local practices of caring for the ill and burying the dead, 2) developing a dedicated ETU in Foya that could efficiently accommodate increases in new admissions, 3) establishing a local hotline and outreach teams from Médecins Sans Frontières (MSF) and local health offices to rapidly transport persons with Ebola-like symptoms to the Foya ETU and safely bury persons suspected of dying from Ebola, 4) establishing a dedicated laboratory facility for rapid case identification, 5) active case-finding in areas with newly reported cases, and 6) training general community health volunteers to conduct contact tracing of persons with known exposures. No cases were reported in the county during April 9–May 31, but cases reappeared in early June (5). The intensity and thoroughness of activities increased in response to the resurgence in Ebola.

In September 2014, national situation reports suggested a decrease in new cases of Ebola in Lofa County. In early October, MoHSW asked CDC to further characterize trends in Ebola in the county. The following data from June 8 to November 1, 2014, were reviewed: 1) aggregate data for newly reported suspected, probable, and confirmed cases of Ebola; 2) case-based data for persons admitted to the Foya ETU operated by MSF; and 3) test results for oral swab specimens collected from persons who died in the community and whose deaths were investigated for possible Ebola.

Aggregate data for newly reported cases were obtained from the county health office and publicly available national situation reports published by Liberian MoHSW. These data include new cases reported daily by local health offices in the six districts of Lofa County. The weekly number of new cases increased from 12 in the week ending June 14 to 153 in the week ending August 16, and then decreased, reaching four new reported cases in the week ending November 1 (Figure 1).

MSF provided deidentified case-based data of persons admitted to the Foya ETU. Final epidemiologic classification of cases was consistent with case definitions described by the World Health Organization (6). Laboratory tests for Ebola virus were performed by the European Mobile Laboratory (EMLab) Consortium in Guéckédou, Guinea, and Foya, Liberia. Reverse transcription–polymerase chain reaction (RT-PCR) assay was used for laboratory confirmation. An illness in a person who tested negative for Ebola virus after more than 72 hours of symptoms was designated as not Ebola, and an illness in a person who tested positive for Ebola virus, regardless of duration of symptoms, was classified as a confirmed case of Ebola. An illness in a person without accompanying laboratory data was designated as an unknown disease.

Case-based data for persons admitted to the Foya ETU describe a trend (Figure 2) similar to that of the aggregate data for newly reported cases. The number of persons admitted increased from 14 in the week ending June 14, to a peak of 133 in the week ending August 16. Admissions then decreased, reaching one person admitted in the week ending November 1. The percentage of persons who had a final classification of not infected with Ebola increased from 25% during June 8–August 9, to a peak of 59% in the week ending August 16, and subsequently decreased to 41% during August 17–November 1. Overall, 40% of persons admitted to the Foya ETU had illnesses not caused by Ebola virus.

Oral swab specimens were collected by outreach teams from MSF and district health offices from persons who died in communities with symptoms suggestive of Ebola. Specimens were analyzed by the EMLab field laboratories using RT-PCR, which has similar performance on oral swab specimens and blood specimens (7). Test results for oral swab specimens from EMLab were linked to case-based data for community decedents from MSF. The trend in the proportion of deaths in the community attributed to Ebola virus also suggested a recent decrease in transmission (Figure 3). During June 8–August 16, a total of 35 (95%) of 37 swab specimens tested positive for Ebola virus; during August 24–November 1, only 21 (25%) of 85 tested positive.

## Discussion

The trends in numbers of newly reported cases, persons admitted to the Foya ETU, and positivity rate among community decedents evaluated for Ebola virus during June 8–November 1, 2014, are consistent with a substantial decrease in transmission of Ebola virus in Lofa County beginning as early as August 17, 2014. The aggregate data from the Lofa County Health Office and case-based data from the Foya ETU describe a peak of reported cases and new admissions respectively in the week ending August 16 followed by a decline in subsequent weeks. The high percentage of positive specimens collected from community decedents during June 8–August 16 suggests that Ebola was causing deaths in communities, whereas the lower percentage during August 24–November 1 suggests that other endemic diseases, such as malaria or typhoid, had become the main causes of mortality as transmission of Ebola virus decreased. The findings from this analysis might indicate the first example in Liberia of a successful strategy to reduce the transmission of Ebola virus in a county with high cumulative incidence.

Transparency in activities and engagement with the community were central to the response strategy in Lofa County. For example, the Foya ETU was designed without high, opaque walls to minimize fear of the facility. Family members were permitted to visit their loved ones in the ETU, either by talking with them across a fence or inside the ward while wearing full personal protective equipment. Decedents in the ETU were buried in the presence of family members at designated burial sites in graves with clear identification. In communities, rapid transport of ill persons to the ETU and safe burial of persons suspected of dying from Ebola demonstrated to the local population that partners could quickly respond to requests for help. During safe burials of community decedents, family members were invited to hold grieving ceremonies according to local customs in memory of the deceased. Engagement with the local population might have built confidence in response activities and contributed to the success of the strategy.

Data on final classifications of patients admitted to the Foya ETU and test results from community decedents indicate ongoing engagement from the community. The high percentage of non-Ebola cases among persons admitted to the ETU during the peak of admissions suggests that the community and health workers were aware that persons with symptoms suggestive of Ebola should be evaluated at the ETU. The high percentage of non-Ebola cases among new admissions in recent weeks and the increasing number of weekly specimens collected from community decedents suggests that trust in response activities remains strong in the local population despite the recent decrease in cases.

Although transmission in Lofa County might have decreased, situation reports from MSF, EMLab, and the World Health Organization have indicated an increase in cases during September and October in Macenta (8,9), a health district in Guinea bordering Lofa County. Based on interviews with response partners, the decrease in cases and community deaths from Ebola in Lofa County is not believed to have resulted from the emigration of ill persons from Lofa to Macenta. However, ill persons are entering Lofa County from elsewhere. Among patients admitted to the Foya ETU in the week ending October 4, half were persons who had exposures in Monrovia, Liberia, before traveling to Lofa County. Expansion of control activities attentive to the needs and sensitivities of the local population in other regions is needed to accelerate progress in stopping the spread of Ebola virus.

Recent reports indicate that transmission of Ebola virus in Liberia is ongoing (10). The findings from this analysis suggest that transmission might be controlled at the county level by a comprehensive response strategy developed by government health offices, nongovernmental organizations, and technical agencies in collaboration with the local population. The strategy in Lofa County might serve as a model for decreasing transmission of Ebola virus in areas where Ebola is still prevalent and spreading. Although transmission of Ebola virus might have decreased, new cases continue to be reported by the Lofa County Health Office and admitted to the Foya ETU. Partners in the response should remain vigilant and continue their activities to further enhance the control of Ebola in Lofa County.

What is already known on this topic?

Lofa County in Liberia has one of the highest numbers of reported cases of Ebola virus disease (Ebola) in West Africa. Government health offices, nongovernmental organizations, and technical agencies coordinated response activities to reduce transmission of Ebola in Lofa County. The intensity and thoroughness of activities increased in response to the resurgence of Ebola in early June.

What is added by this report?

Trends in new reported cases, admissions to the dedicated Ebola treatment unit in the town of Foya, and test results of community decedents evaluated for Ebola virus suggest transmission of Ebola virus decreased in Lofa County as early as August 17, 2014, following rapid scale-up of response activities after a resurgence of Ebola in early June.

What are the implications for public health practice?

A comprehensive Ebola response strategy developed with participation from the local community and rapidly scaled up following resurgence of Ebola might have reduced the spread of Ebola virus in Lofa County. The strategy implemented in Lofa County might serve as a model for reducing transmission of Ebola virus in other affected areas.

## Figures and Tables

**FIGURE 1:**
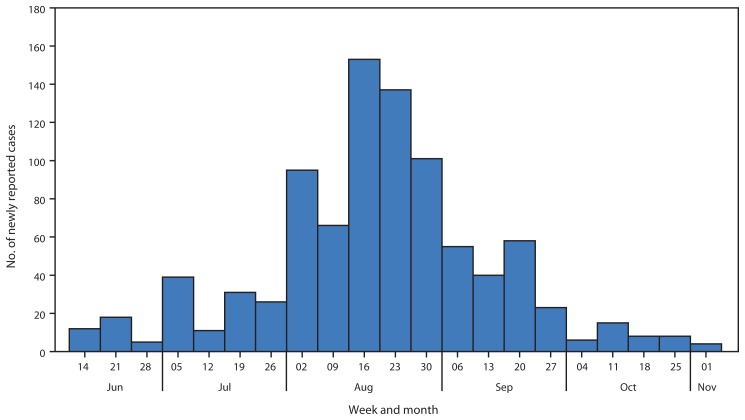
Aggregate number of newly reported Ebola cases, by week — Lofa County, Liberia, June 8–November 1, 2014

**FIGURE 2:**
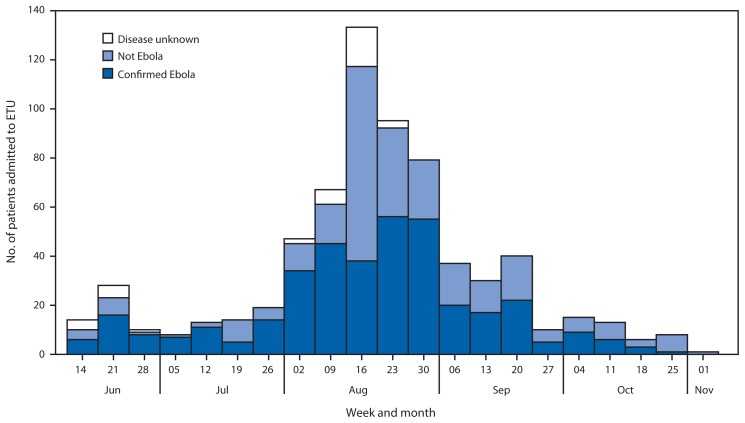
Number of patients admitted to an Ebola Treatment Unit (ETU) operated by Médecins Sans Frontières in the town of Foya, by week and final classification — Lofa County, Liberia, June 8–November 1, 2014

**FIGURE 3:**
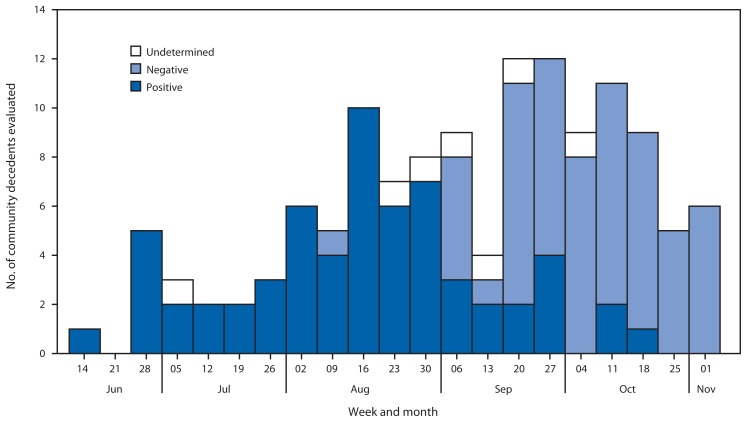
Test results for specimens collected from community decedents evaluated for Ebola, by week — Lofa County, Liberia, June 8–November 1, 2014
